# Chemokine Co-Receptor CCR5/CXCR4-Dependent Modulation of Kv2.1 Channel Confers Acute Neuroprotection to HIV-1 Glycoprotein gp120 Exposure

**DOI:** 10.1371/journal.pone.0076698

**Published:** 2013-09-24

**Authors:** Andrew J. Shepherd, Lipin Loo, Durga P. Mohapatra

**Affiliations:** 1 Department of Pharmacology, Roy J. and Lucille A. Carver College of Medicine, The University of Iowa, Iowa City, Iowa, United States of America; 2 Department of Anesthesia, Roy J. and Lucille A. Carver College of Medicine, The University of Iowa, Iowa City, Iowa, United States of America; University of Texas Health Science Center, United States of America

## Abstract

Infection with human immunodeficiency virus-1 (HIV-1) within the brain has long been known to be associated with neurodegeneration and neurocognitive disorder (referred as HAND), a condition characterized in its early stages by declining cognitive function and behavioral disturbances. Mechanistically, the HIV-1 coat glycoprotein 120 (gp120) has been suggested to be a critical factor inducing apoptotic cell death in neurons via the activation of p38 mitogen-activated protein kinase (MAPK), upon chronic exposure to the virus. Here we show that acute exposure of neurons to HIV-1 gp120 elicits a homeostatic response, which provides protection against non-apoptotic cell death, involving the major somatodendritic voltage-gated K^+^ (Kv) channel Kv2.1 as the key mediator. The Kv2.1 channel has recently been shown to provide homeostatic control of neuronal excitability under conditions of seizures, ischemia and neuromodulation/neuroinflammation. Following acute exposure to gp120, cultured rat hippocampal neurons show rapid dephosphorylation of the Kv2.1 protein, which ultimately leads to changes in specific sub-cellular localization and voltage-dependent channel activation properties of Kv2.1. Such modifications in Kv2.1 are dependent on the activation of the chemokine co-receptors CCR5 and CXCR4, and subsequent activation of the protein phosphatase calcineurin. This leads to the overall suppression of neuronal excitability and provides neurons with a homeostatic protective mechanism. Specific blockade of calcineurin and Kv2.1 channel activity led to significant enhancement of non-apoptotic neuronal death upon acute gp120 treatment. These observations shed new light on the intrinsic homeostatic mechanisms of neuronal resilience during the acute stages of neuro-HIV infections.

## Introduction

According to the United Nations Programme on human immunodeficiency virus (HIV) and acquired immunodeficiency syndrome (AIDS), approximately 34 million people Worldwide are infected with HIV [1]. Aside from the devastating immunological effects of the resultant AIDS, as many as 40% of HIV-positive patients suffer from varying degrees of neuro-viral infection and subsequent HIV-associated neurodegeneration and neurocognitive disorder (HAND), which can lead to cognitive decline and diminished quality of life [2]. Highly-active antiretroviral therapy (HAART) is largely responsible for the recent decrease in incidence of HIV-associated dementia, the most advanced form of HAND [3]. However, increased life expectancy of HIV patients and emergence of HIV strains resistant to HAART therapy, coupled with the persistence of latent reservoirs of infection within the central nervous system account for the increasing prevalence of HAND [2,3]. This necessitates further exploration of cellular mechanisms and development of novel therapeutic approaches that could provide effective neuroprotection.

The HIV-1 coat glycoprotein gp120 has been reported to induce cellular injury and apoptosis in neurons upon sustained exposure both *in vitro* and *in vivo*, and is thought to contribute to the pathogenesis of HAND [4-9]. In addition to the surface glycoprotein CD4, gp120 also binds to the chemokine receptors CCR5 and CXCR4, which are expressed by neurons, astrocytes and microglia [7,10-12]. This ligand-receptor interaction (either directly or via *trans*-signaling) leads to transient and oscillatory, as well as sustained elevation of intracellular Ca^2+^ ([Ca^2+^]_i_) within neurons [5,7,11,13-17], potentially leading to enhanced action potential firing and increased neuronal excitation. In fact, it has been reported that the endogenous chemokine ligands for CXCR4, stromal cell-derived factor 1-alpha (SDF-1α), and for CCR5, regulated on activation, normal T cell expressed and secreted (RANTES) elicit substantial [Ca^2+^]_i_ in neurons [8,10,14], which subsequently activates the Ca^2+^/calmodulin-dependent protein phosphatase 2B or calcineurin [8,18]. This leads to dephosphorylation of multiple proteins in mammalian brain neurons, including the principal voltage-gated K^+^ (Kv) channel Kv2.1 [8], which is the major constituent of total delayed-rectifier K^+^ currents (*I*_DR_) [19-21]. Kv2.1 is expressed as a constitutively hyperphosphorylated protein and localized in the form of surface clusters in the somata and proximal dendrites of most mammalian central neurons [22]. Calcineurin-dependent dephosphorylation of Kv2.1 disrupts this clustered localization and enhances the voltage-dependence of channel activation in a hyperpolarizing direction [21,23-26], such that action potential firing frequency is limited [21,27]. This phenomenon has been shown to underlie homeostatic suppression of hyperexcitability in response to excitotoxic stimuli such as seizures and ischemia, as well as under neuromodulatory and neuroinflammatory conditions [21,28,29].

We hypothesized that, immediately following the exposure of neurons to HIV-1 gp120 and the resultant increase in [Ca^2+^]_i_ [5,10,11,13,14,17], Kv2.1 would undergo similar acute calcineurin-dependent changes in an attempt to homeostatically suppress membrane excitability and provide neuroprotection. Here we show that acute exposure of hippocampal neurons to gp120 led to elevated [Ca^2+^]_i_ in rat hippocampal neurons, as well as rapid dephosphorylation and disruption of clustered localization of Kv2.1 protein, changes that are dependent on CCR5/CXCR4 co-receptor signaling and activation of calcineurin. These changes led to significant enhancement of the voltage-dependent activation of *I*_DR_ at near resting membrane potentials, by specific enhancement of currents through the Kv2.1 channel. Accordingly, pharmacological blockade of Kv2.1 led to increased non-apoptotic cell death in neurons acutely exposed to gp120, which otherwise did not influence neuronal cell death. Results from this study emphasize the crucial role of Kv2.1 in providing early neuroprotection to HIV-1 gp120, and potentially other neurotropic viral infections.

## Materials and Methods

All experiments involving the use of rats, and the procedures followed therein were carried out in strict accordance with the recommendations in the Guide for the Care and Use of Laboratory Animals of the National Institutes of Health. The animal use and care protocols (1103053 and 1109203) were approved by the University of Iowa Institutional Animal Care and Use Committee. All surgery and euthanasia were performed under isoflurane anesthesia, and every effort was made to minimize the number of rats used for experiments and their suffering.

### Chemicals and reagents

Recombinant mouse SDF-1α, RANTES, WZ811, Maraviroc, FK506, MK801, and SB203580 were purchased from R&D Systems – Tocris Bioscience (Minneapolis, MN); purified recombinant HIV-1 gp120 IIIB was from ImmunoDiagostics (Woburn, MA); stromatoxin-1 (ScTx-1), trypsin, and glutamate were from Sigma-Aldrich (St. Louis, MO); and Fura-2-AM, Hank’s balanced salt solution (HBSS), B27 growth supplement, Dulbecco’s modified Eagle’s medium (DMEM), GlutaMAX, penicillin/streptomycin and Neurobasal media were from Invitrogen (Life Technologies, Grand Island, NY). All other chemicals used in this study were purchased from Sigma, Roche Applied Science, Bio-Rad, Fisher Scientific and VWR. The NeuroMab antibodies were purchased from the UC Davis/NIH NeuroMab Facility through Antibodies Inc., Davis, CA.

### Primary culture of rat hippocampal neurons

Hippocampal neurons from rat embryos were isolated and cultured as described previously [8,30]. Briefly, hippocampi from E18 rat embryos of either sex (Sprague-Dawley; Harlan) were removed, washed and subjected to trypsin digestion (1 mg/ml), followed by centrifugation and resuspension of dissociated cells in Neurobasal medium containing B27, 0.6 mM glutamine, and 5% horse serum. Cells were plated onto poly-L-lysine-coated 35 mm tissue culture-grade dishes for biochemical experiments (density -200,000 cells per 35 mm dish), as well as onto poly-L-lysine-coated glass coverslips for immunocytochemical, ratiometric Ca^2+^ imaging and electrophysiological experiments (density -100,000 cells per 35 mm dish). The cells were then incubated at 37°C for 3–4 h in a humidified incubator with 5% CO_2_, after which the medium was replaced with serum-free Neurobasal media containing B27 growth supplement and L-glutamine, and maintained at 37°C in a humidified incubator with 5% CO_2_. One-third of the medium was exchanged weekly with fresh new media. All experiments were performed on neurons that were cultured for 14 to 17 days *in vitro* (DIV).

### Ratiometric Ca^2+^ imaging

Functional Ca^2+^ imaging on cultured rat hippocampal neurons was performed as described previously [8,31]. Neurons on glass coverslips were incubated at room temperature (22°C) for 30 min with 5 µM of Fura-2-AM. The coverslip was then placed in the recording chamber mounted on the stage of an inverted IX-71 microscope (Olympus) and perfused for 10 min with the standard extracellular HEPES-buffered Hank’s salt solution (HH buffer) composed of the following (in mM): 140 NaCl, 5 KCl, 1.3 CaCl_2_, 0.4 MgSO_4_, 0.5 MgCl_2_, 0.4 KH_2_PO_4_, 0.6 NaHPO_4_, 3 NaHCO_3_, 10 glucose, 10 HEPES, pH 7.4, with NaOH (310 mOsm/kg with sucrose). Fluorescence was alternately excited at 340 nm and 380 nm (both 12 nm band pass) using the Polychrome IV monochromator (T.I.L.L. Photonics), via a 20X objective (NA 0.75; Olympus). Emitted fluorescence was collected at 510 (80) nm using an IMAGO CCD camera (T.I.L.L. Photonics). Pairs of 340/380 nm images were sampled at 2 Hz. Bath application of gp120 (1 nM or 10 nM, 450 s) and 50 mM KCl (‘K50,’ 200 s) was carried out in HH buffer. The fluorescence ratio (*R* = F_340_/F_380_) values over time were processed and analyzed using TILLvisION 4.0.1.2 (T.I.L.L. Photonics) and Origin 7.0 (Origin Lab) software, as described previously [8].

### Biochemical analysis of Kv2.1 proteins in hippocampal neurons

SDS-PAGE (7.5%) and immunoblotting of 1% Triton X-100-soluble protein extracts from cultured rat hippocampal neurons (15-16 DIV; without or with drug treatments) were performed as described previously [8,24,29,30]. For experiments with alkaline phosphatase (AP) treatment, aliquots of neuronal lysates were incubated with calf intestinal AP (100 U/ml; Roche) in lysis buffer containing 0.1% SDS for 2 h at 37°C. As a control, lysate aliquots without AP were incubated for 2 h at 37°C. Following gel transfer, nitrocellulose membranes were first incubated with the blocking solution [4% fat-free milk powder in Tris-buffered saline (TBS)] and subsequently incubated with mouse monoclonal anti-Kv2.1 antibody (1:1000; clone K89/34 from NeuroMab). After washing 3 times with the blocking solution, blots were incubated with horseradish peroxidase (HRP)-conjugated anti-mouse IgG antibody (1:10,000, Antibodies, Inc., Davis, CA). Blots were developed using enhanced chemiluminescence reagent (PerkinElmer), and immunoreactive bands were visualized by exposure to X-ray film (BioMax, Kodak). X-ray signal intensities of immunoreactive bands were quantified using the NIH ImageJ software as detailed earlier [8]. All quantifications were performed on a minimum of three independent samples.

### Immunocytochemical staining of cultured neurons

Immunocytochemical staining of cultured rat hippocampal neurons was performed as described previously [8,30]. Neurons (14-17 DIV) cultured on glass coverslips were fixed for 30 min at 4°C with 4% ice-cold paraformaldehyde (PFA) and 4% sucrose in 0.1 M phosphate buffer (PB), pH 7.3. Neurons were then permeabilized and incubated in the blocking solution (4% fat-free milk powder in TBS containing 0.1% Triton X-100) for 1 h at room temperature. The coverslips were then incubated with the primary antibodies [rabbit polyclonal anti-MAP2 (1:1000; Sigma), and mouse monoclonal anti-Kv2.1 (1:1000; clone K89/34 from NeuroMab); rabbit polyclonal Alexa Fluor-488-conjugated anti-cleaved-caspase-3 (1:100; Cell Signaling)] in the blocking solution for 1 h at room temperature. After washing 3 times with the blocking solution, coverslips were incubated with species-specific Alexa Fluor 488-, 555- and 633-conjugated secondary antibodies (1:2,000) in the blocking solution for 1 h at room temperature. Coverslips were then washed and mounted onto glass slides with ProLong Gold antifade mounting medium. Immunofluorescence images were captured by an MRc-5 digital camera connected to a Zeiss AxioImager epifluorescence microscope, using AxioVision software (Carl Zeiss Microscopy LLC, Thornwood, NY). Images were taken with a 63X Plan-Apochromat objective (NA 1.4; Zeiss; for Kv2.1 localization experiments) or with a 10X Plan-Apochromat objective (NA 0.25; Zeiss; for cleaved-caspase-3 experiments). All the images were transferred to Photoshop software (Adobe Systems, San Jose, CA) as TIFF files. For quantification of the percentage of neurons with a specific localization pattern of Kv2.1, slides were coded such that counting was conducted in a blinded fashion. Cells expressing Kv2.1 were divided into 3 typical sub-cellular localization groups: ‘clustered’, in which the vast majority of Kv2.1 immunoreactivity is confined to distinct surface clusters; ‘intermediate’, in which some Kv2.1 clustering remains, but there is also a substantial pool of dispersed Kv2.1 staining in the same cell; and ‘dispersed’, in which all or the vast majority of Kv2.1 immunoreactivity is dispersed across the plasma membrane surrounding the soma and proximal dendrites. Results were obtained by counting ≥250 cells for each treatment condition, from four or more independent batches of cultures. Data are presented as mean ± SEM of relative localization groups.

### Patch-clamp electrophysiology and data analysis

Currents were recorded from cultured rat hippocampal neurons at room temperature with the whole-cell configuration of the patch-clamp technique, as described earlier [8,21]. Patch pipettes were pulled from borosilicate glass tubes (TW150F-4, World Precision Instruments, Sarasota, FL) and heat-polished at the tip using a microforge (MF200, WPI) to give a resistance of 3–6 MΩ when filled with the pipette solution consisting of (in mM): 140 KCl, 5 NaCl, 1 CaCl_2_, 1 MgCl_2_, 10 HEPES, 10 EGTA and 0.4 Na-ATP, pH 7.3. The extracellular buffer consisted of (in mM): 140 NaCl, 5 KCl, 2 CaCl_2_, 1 MgCl_2_, 10 HEPES and 10 glucose, pH 7.4 with, 0.5 µM tetrodotoxin (TTX; to block fast-activating Nav currents). Cells were treated with purified recombinant HIV-1 gp120 in culture media for 30 min at 37°C, without or with ScTx-1, immediately before the beginning of recordings. ScTx-1 was also added to the extracellular buffer for the duration of the recording. Currents were recorded with an Axopatch 200B patch-clamp amplifier connected to a Digidata 1440A data acquisition system (Molecular Devices, Sunnyvale, CA), with a sampling rate of 10 kHz and filtering at 2 kHz using a digital Bessel filter. pCLAMP 10 software (Molecular Devices) was used for the acquisition of currents, and Clampfit 10 (Molecular Devices) and Origin 7.0 (Microcal) software were used for the analysis of currents and preparing traces/figures. For voltage-dependent activation experiments on neuronal *I*_DR_, cells were held at -100 mV and step depolarized to +80 mV for 250 ms in +10 mV increments. A pre-pulse at +10 mV for 40 ms was given before each test pulse to inactivate the majority of *I*_A_. All currents were capacitance and series resistance compensated, and leak subtractions were performed offline. The Nernst K^+^ equilibrium potential (*E*_K_) was calculated as -85 mV. Peak outward currents from the last 150 ms of depolarizing test pulses were taken as *I*_DR_, and the current density was calculated by dividing the peak currents (pA) at each individual depolarizing test pulse by the cell capacitance (pF). Voltage-dependent activation/conductance curves were generated as described previously [8,21], and the half-maximal voltage-dependent activation potentials/conductances (*G*_1/2_) are mentioned in the respective figures. Data are presented as means ± SEM or fitted value ± SE of the Boltzman’s equation fit for the conductance-voltage (*G-V*) curves.

### Immunocytochemistry-based cell death assay

For the quantification of cell death, an immunofluorescence-based Live-Dead cell viability assay kit (Invitrogen) was used, as described earlier [8], following manufacturer’s instructions with slight modifications. Briefly, cells were incubated with 1 µM ethidium homodimer-1 (EthD-1) in 0.1 M PBS, pH 7.4 for 30 min after various drug treatments and immediately prior to fixation with PFA. Cells were then permeabilized and blocked with 4% fat-free milk powder dissolved in TBS containing 0.1% Triton X-100 for 1 h at room temperature, followed by incubation with rabbit polyclonal anti-MAP2 antibody (1:1000; Sigma) 1 h at room temperature. After washing 3 times with the blocking solution, cells were incubated with Alexa Fluor 488-conjugated anti-rabbit IgG secondary antibody (1:2,000) for 1 h at room temperature. After mounting onto slides, immunofluorescence images were captured by an MRc-5 digital camera connected to a Zeiss AxioImager epifluorescence microscope (Carl Zeiss). Images were taken with a 10X Plan-Apochromat objective (NA 0.25; Carl Zeiss). All the images were transferred to Photoshop software (Adobe Systems, San Jose, CA) as TIFF files. Cell counting was conducted in a blinded fashion, taking into account only MAP 2-positive cells (neurons). Results were obtained by counting >650 cells for each treatment condition, from four or more independent batches of neuronal cultures. Data are presented as mean ± SEM.

### Statistical analysis

Data were analyzed using Student’s t-test and one-way analysis of variance (ANOVA) with post-hoc Bonferroni’s correction, in order to test the statistical significance of the differences between various treatments. *p*<0.05 in each set of data comparisons were considered statistically significant. All statistical analyses were conducted using SPSS-21 software (IBM, Armonk, NY).

## Results

### Acute exposure of hippocampal neurons to HIV-1 gp120 elevates intracellular calcium concentrations and dephosphorylation and altered localization of Kv2.1

**Figure 1:**
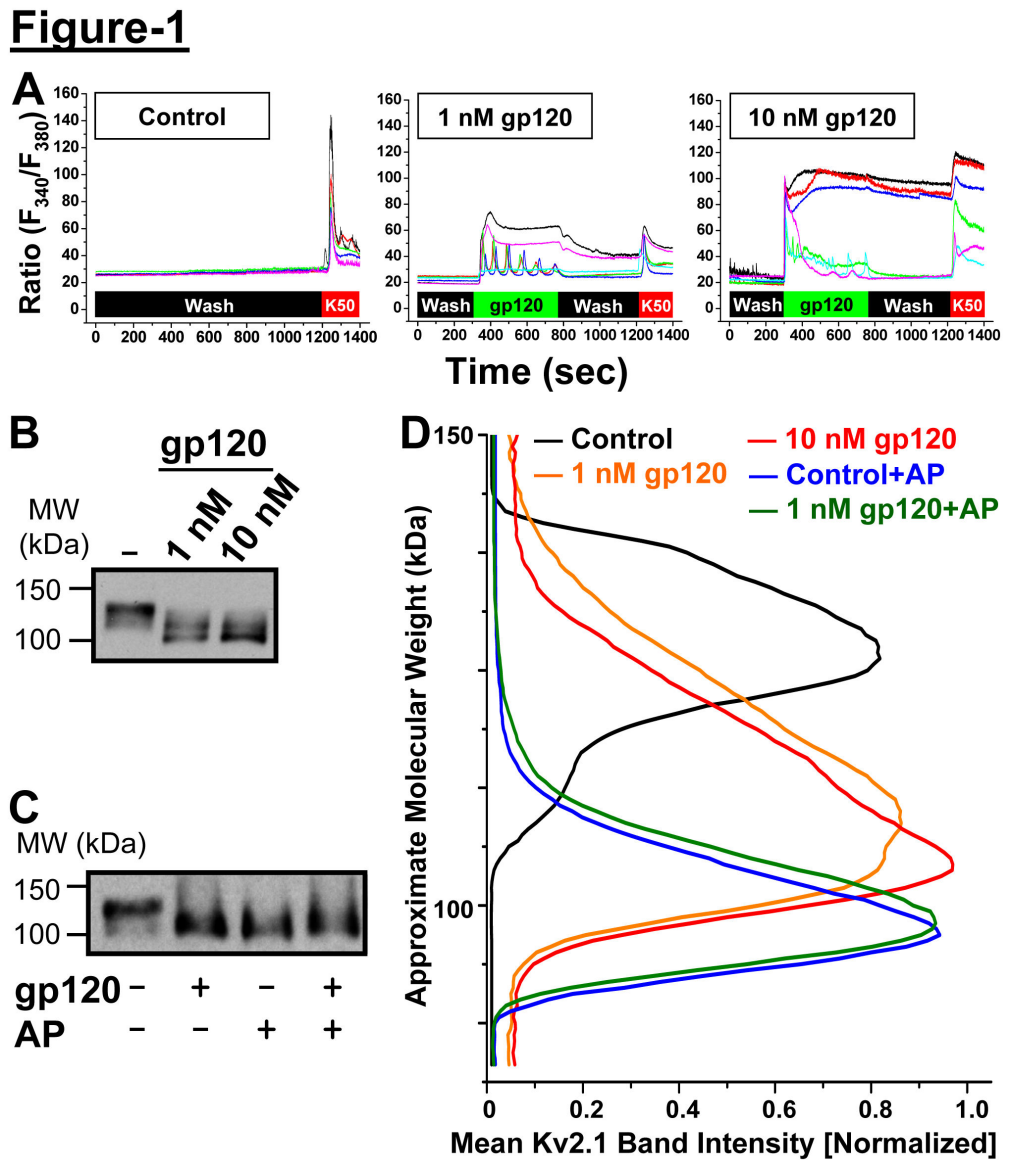
Acute HIV-1 gp120 induces calcium (Ca^2+^) flux and dephosphorylation of Kv2.1 in hippocampal neurons. ***A***, Representative traces of ratiometric Ca^2+^ imaging in cultured rat hippocampal neurons using Fura-2. In the left panel, HH buffer (wash) was perfused over the coverslip, prior to a pulse of 50 mM KCl (K50) to demonstrate depolarization-induced Ca^2+^ influx in each neuron. Following exposure to 1 nM gp120 (center panel), neurons demonstrate transient or sustained changes in the fluorescence ratio (340: 380 nm), an indicator of increased intracellular Ca^2+^. This effect was partially washed out upon washing with HH buffer. With 10 nM gp120 (right panel), the magnitude of Ca^2+^ influx is increased, such that several neurons show sustained elevations in [Ca^2+^]_i_ that persist for minutes after gp120 perfusion has ended. ***B***, Immunoblot analysis of Kv2.1 phosphorylation levels upon acute gp120 treatment (1 and 10 nM, 30 min) of cultured rat hippocampal neurons, showing increased electrophoretic mobility of Kv2.1 immunoreactive bands in a dose-dependent manner. This apparent decrease in molecular weight can be attributed solely to channel dephosphorylation, since alkaline phosphatase (AP) treatment of samples (shown in ***C***) decreases the molecular weight of the channel protein to the same, minimal level as predicted from the deduced amino acid sequence of Kv2.1. Numbers on the left in panels ***B*** & ***C*** denote the approximate molecular weight. ***D***, Quantification of the extent of Kv2.1 phosphorylation upon gp120 exposure as shown in panels ***B*** & ***C***). Densitometric analysis of Kv2.1 immunoreactive band intensities at molecular weight range of ~90 to ~150 kDa (see methods for details), showing phospho-Kv2.1 with peak intensity at ~130 kDa and dephospho-Kv2.1 with peak intensity at ~100 kDa. Data are presented as mean (n = 4-5 for each group).

Prolonged exposure of neurons to HIV-1 gp120 has been shown to induce apoptotic cell death in neurons, thereby triggering the well-known clinical manifestations of neurodegeneration and associative cognitive deficits [4-7]. In cell-based experimental investigations, application of gp120 has been shown to induce rapid, oscillatory and sustained elevation of [Ca^2+^]_i_ (for several minutes) in cultured rodent hippocampal and cortical neurons [5,10,11,13,14,17]. In the subsequent hours/days, neurons undergo apoptosis mediated mainly by p38 mitogen-activated protein kinase (p38 MAPK) [5]. However, the role of such [Ca^2+^]_i_ elevation in neuronal function and/or death remains unexplained. Moreover, such elevations in [Ca^2+^]_i_ are not at a magnitude that could induce rapid necrotic death in neurons. We have previously shown that rapid elevation of neuronal Ca^2+^ levels by increased excitatory activity, neuromodulatory and neuroinflammatory stimuli lead to the activation of calcineurin [8,23,24,29]. Thus, we first established that acute exposure of cultured rat hippocampal neurons to gp120 was prompting similar elevation in [Ca^2+^]_i_. Using the ratiometric Ca^2+^-sensitive dye Fura-2, we show that untreated neurons exhibit a stable 340: 380 nm fluorescence ratio, indicative of low resting levels of [Ca^2+^]_i_; however, treatment with 1 nM or 10 nM gp120 caused elevations in [Ca^2+^]_i_ in most neurons tested in a dose-dependent manner (Figure 1A). Consistent with prior observations [32], neuronal responses could be subdivided into either a single peak or an oscillatory response, many of which recovered to baseline in the presence of gp120 (Figure 1A). In addition, the rapid recovery to near baseline levels of [Ca^2+^]_i_ in the majority of neurons, coupled with the robust depolarization seen upon exposure to 50 mM KCl suggest that acute gp120 application at these doses is not toxic under these circumstances.

Given that gp120 elicits increased [Ca^2+^]_i_, we next investigated whether this could lead to dephosphorylation of the major somatodendritic Kv channel, Kv2.1, via activation of the Ca^2+^/calmodulin-dependent protein phosphatase calcineurin [21,25,26]. In hippocampal neurons Kv2.1 is expressed as a constitutively hyper-phosphorylated protein [23,25]. To assess and quantify the extent of Kv2.1 phosphorylation state, altered electrophoretic mobility of the channel protein in SDS-PAGE-immunoblot analysis is utilized frequently and most reliably, as reported earlier [8,23,24,26]. Such dephosphorylation of Kv2.1 channel protein leads to an increase in *I*_DR_ conductance, which limits neuronal excitability, ultimately providing a neuroprotective mechanism [21,24,29]. Cultured rat hippocampal neurons were treated with gp120 (1 and 10 nM, 30 min) and the lysates were analyzed by immunoblotting, in which the Kv2.1 protein showed a significant increase in the electrophoretic mobility, indicating channel dephosphorylation, as compared to untreated conditions, where a majority of Kv2.1 is constitutively hyper-phosphorylated (Figure 1B-C). Upon phosphatase digestion of cultured neuron lysates with AP (100 U/ml for 2 h at 37°C), the Kv2.1 channel protein undergoes near-complete dephosphorylation, as evidenced by shift in the electrophoretic mobility of Kv2.1 immunoreactive band that matches with the molecular weight from deduced amino acid sequence of the protein (~97 kDa, Figure 1C-D). Furthermore, both control and gp120-treated samples are reduced to the same apparent molecular weight upon AP digestion (Figure 1B-C). This suggests that the gp120-induced Kv2.1 protein dephosphorylation is in line with increased [Ca^2+^]_i_ and enhanced electrophoretic mobility of the channel protein, consistent with what has been observed earlier for chemokine SDF-1α and glutamate treatment of neurons [8,23]. Under these conditions a relatively lesser degree of protein dephosphorylation is observed in comparison to AP treatment. It is important to note here that gp120/glutamate/SDF-1α only induces Ca^2+^-dependent dephosphorylation of Kv2.1, which does not induce complete dephosphorylation of the channel protein, whereas AP treatment leads to complete (or near complete) removal of phospho-modifications in the protein *in vitro*. Also, AP treatment of the lysates from neurons that are exposed to gp120 led to a further small downward shift in the electrophoretic mobility of Kv2.1 protein, but to an extent almost identical to that observed with AP digested lysates from non-gp120 treated neurons (Figure 1C-D). This clearly and convincingly suggests that gp120-induced shift in the electrophoretic mobility of Kv2.1 immunoreactive band is exclusively due to protein dephosphorylation.

**Figure 2:**
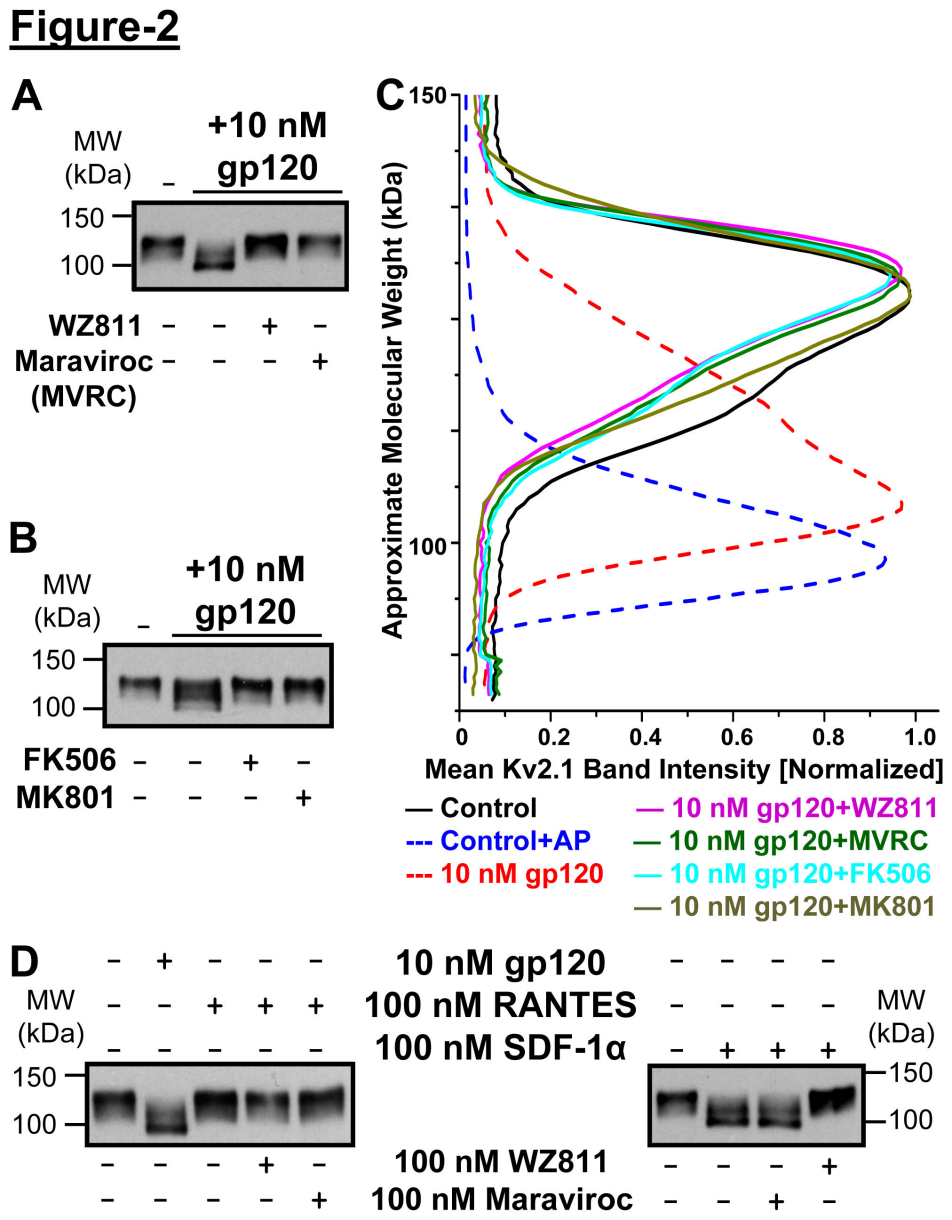
HIV-1 gp120-induced dephosphorylation of Kv2.1 is dependent upon activation of CCR5 and CXCR4, as well as on NMDA receptor-mediated activation of protein phosphatase 2B or calcineurin. ***A***, gp120-induced (10 nM, 30 min) Kv2.1 dephosphorylation was attenuated upon pre/co-application of inhibitors of CXCR4 (WZ811, 100 nM) or CCR5 (Maraviroc or MVRC, 100 nM) to cultured rat hippocampal neurons. ***B***, gp120-induced Kv2.1 dephosphorylation could also be attenuated upon pre/co-application of cultured rat hippocampal neurons with the calcineurin inhibitor FK506 (10 µM), or the NMDAR antagonist MK801 (1 µM). ***C***, Densitometric quantification of the extent of Kv2.1 phosphorylation state upon different drug treatments as shown in panels ***A*** & ***B***. Data are presented as mean (n = 3 for each group). Dotted lines denote the same data traces from Figure 1D plotted for comparison. ***D***, Direct activation of CXCR4, but not CCR5 by their respective endogenous chemokine agonists (SDF-1α and RANTES) led to dephosphorylation of Kv2.1 in cultured rat hippocampal neurons, which was attenuated by the CXCR4 inhibitor WZ811. Numbers on the left in blot panels denote the approximate molecular weight.

It was previously shown that gp120-induced [Ca^2+^]_i_ elevation in neurons is dependent on *trans*-glutamate signaling via GluN-type glutamate receptors [13]. Pre-treatment of neurons with the inhibitor of calcineurin (FK506; 10 µM, 10 min) or GluN (MK801; 10 µM, 10 min) prior to gp120 treatment (10 nM, 30 min) led to the attenuation of Kv2.1 protein dephosphorylation (Figure 2A, C). Since it is well established that gp120 induces [Ca^2+^]_i_ elevation by acting on CCR5/CXCR4 co-receptors [7,13], we next verified whether gp120-induced dephosphorylation of Kv2.1 protein is dependent on the activation of CCR5 and/or CXCR4. Pre-treatment of neurons with the CCR5 inhibitor maraviroc or the CXCR4 inhibitor WZ811 (both 100 nM, 10 min) prior to gp120 treatment (10 nM, 30 min) led to attenuation of Kv2.1 protein dephosphorylation (Figure 2A, C). Recently, we showed that direct activation of CXCR4 by its endogenous chemokine ligand SDF-1α led to Kv2.1 dephosphorylation [8], which was recapitulated here, and could be attenuated by pre-treatment with WZ811, but not by maraviroc (Figure 2B-C). However, treatment of neurons with an endogenous CCR5 chemokine ligand, RANTES (100 nM, 30 min) did not lead to any change in the constitutive phosphorylation state of Kv2.1 protein. These results indicate that gp120-induced dephosphorylation of Kv2.1 protein is dependent on CCR5/CXCR4 co-receptor activation; however, such Ca^2+^/calcineurin-dependent dephosphorylation of the channel protein is exclusively mediated via a CXCR4-mediated downstream signaling component.

**Figure 3:**
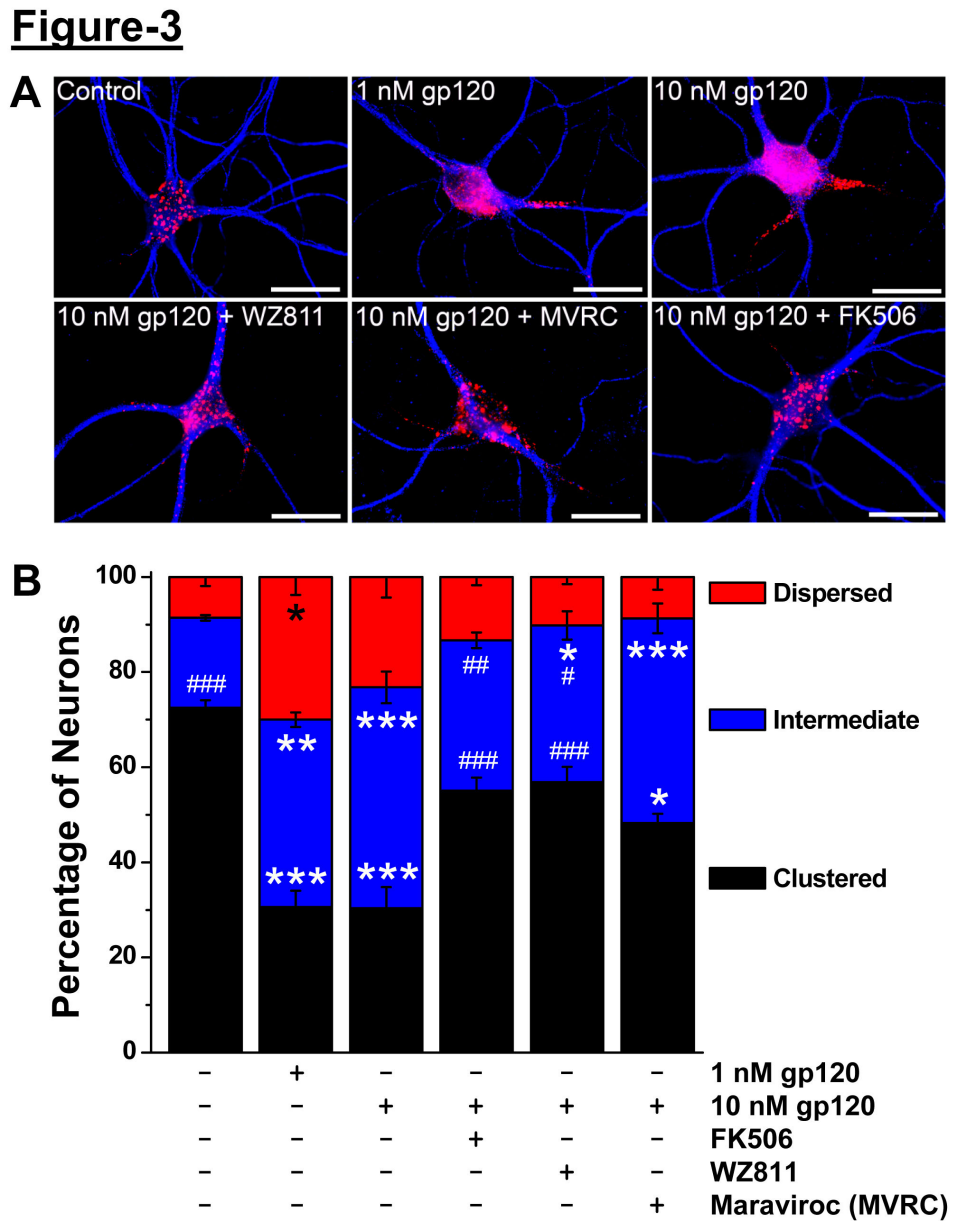
Acute gp120 treatment leads to CCR5/CXCR4/calcineurin-dependent disruption of Kv2.1 channel localization in hippocampal neurons. ***A***, Representative images of cultured rat hippocampal neurons stained with MAP2 (blue) and Kv2.1 (red). Treatment with gp120 (10 nM, 30 minutes) causes dispersal of Kv2.1 clusters when compared to control/untreated conditions, which could be attenuated by pre/co-application with CXCR4 inhibitor WZ811 (100 nM), CCR5 inhibitor Maraviroc (100 nM), and calcineurin inhibitor FK506 (10 µM). Magnification: 63X, scale bar: 25 µm. ***B***, Stacked column plots showing quantification of Kv2.1 distribution in hippocampal neurons. Kv2.1 distribution in neurons either showing fully clustered or fully dispersed or intermediate under different drug treatment conditions (on the bottom of the graph), expressed as mean ± SEM. n = 271, 281, 400, 398, 280, and 363 for control/untreated, 1 nM gp120, 10 nM gp120, 100 nM WZ811 + 10 nM gp120, 100 nM Maraviroc + 10 nM gp120, and 10 M FK506 + 10 nM gp120 treatment groups, respectively, from >3 batches of cultured hippocampal neurons. *p<0.05, **p<0.01 and ***p<0.001 indicate significantly different in comparison to control/untreated conditions; ^#^ p<0.05, ^# #^ p<0.01 and ^# # #^ p<0.001 indicate significantly different in comparison to gp120-treatment conditions (one way ANOVA with post-hoc Bonferroni’s correction).

Kv2.1 protein dephosphorylation is tightly associated with changes in its subcellular distribution, characterized by distinct clustered localization (phosphorylated Kv2.1) to a more dispersed localization (dephosphorylated Kv2.1) throughout the neuronal soma and proximal dendrites [8,23,24]. Consistent with the results from immunoblot analysis (Figures 1, 2), a majority of control neurons stained with anti-Kv2.1 antibody display a clustered distribution, whereas most of the clustered localization is abolished upon treatment with gp120 (10 nM, 30 min), as was observed with SDF-1α treatment [8] (Figure 3A). Acute gp120 treatment prompted a lateral diffusion of Kv2.1 that significantly reduced the population of clustered neurons, shifting the population distribution more to the intermediate and diffuse patterns (Figure 3A-B. Pre-treatment of neurons (for 10 min) with inhibitors of CXCR4 (100 nM WZ811) or CCR5 (100 nM maraviroc) or calcineurin (10 µM FK506) attenuated gp120-induced re-distribution of Kv2.1 channel protein on the soma and proximal dendrites (Figure 3A-B). Dispersal of clustered localization of Kv2.1 upon SDF-1α treatment was also attenuated by pre-treatment with WZ811, but not by maraviroc (not shown). These results suggest that gp120, via activation of CCR5/CXCR4 co-receptors and downstream CXCR4 signaling-mediated activation of calcineurin, leads to rapid dephosphorylation of Kv2.1 protein that results in disruption of clustered localization of the channel protein.

### Acute gp120 enhances *I*_DR_ in hippocampal neurons by specific modulation of Kv2.1

**Figure 4:**
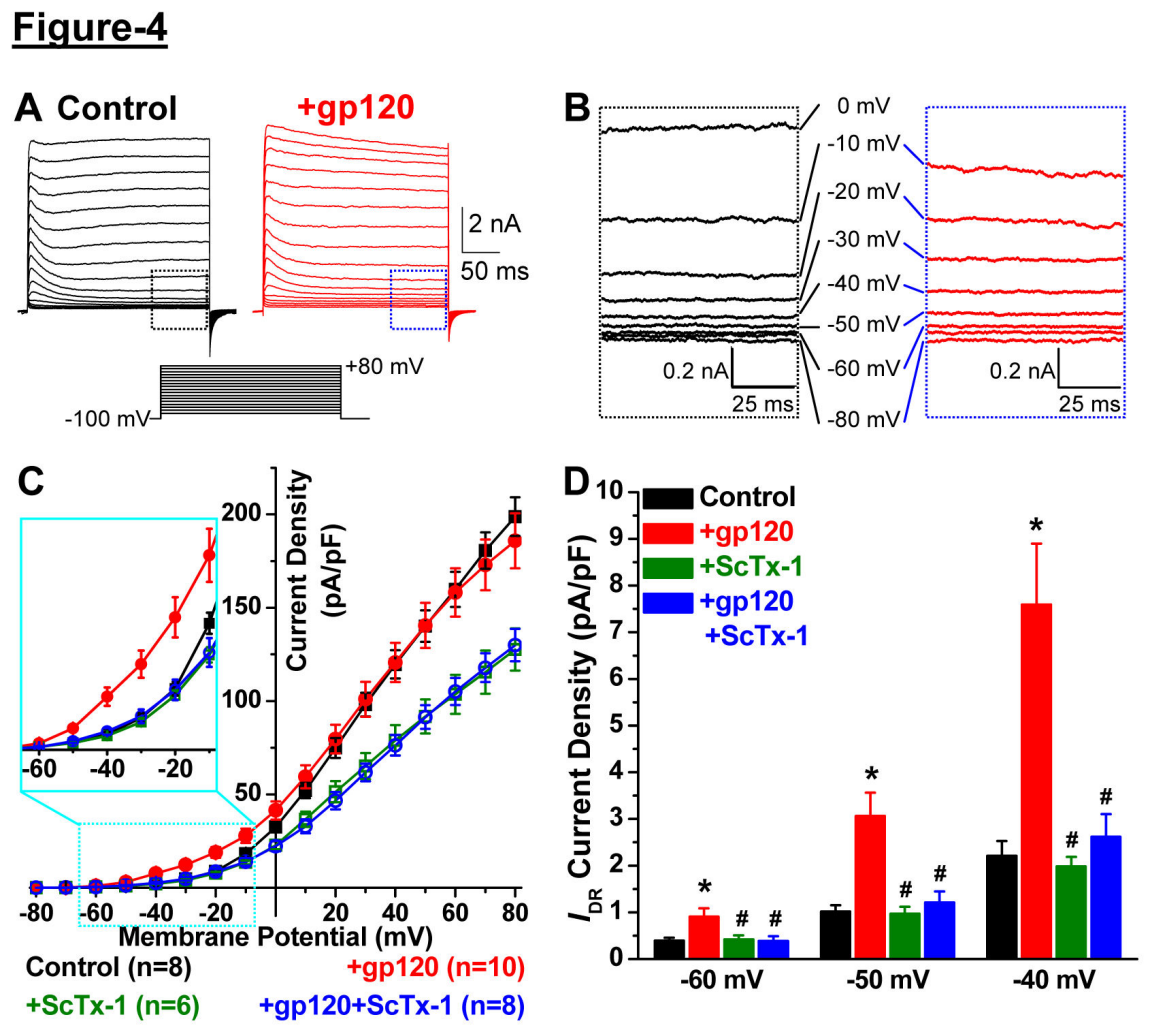
Acute exposure to HIV-1 gp120 enhances neuronal *I*_DR_ current density via specific modulation of the Kv2.1 channel. ***A***, Representative traces of *I*_DR_ currents recorded from cultured rat hippocampal neurons under whole-cell voltage-clamp mode using the shown voltage protocol, without (control) or with gp120 treatment (10 nM, 30 min). Magnified views of current traces within the marked boxes in panel ***A*** (control – black, gp-120 – blue), as well as the corresponding test-potentials are shown in panel ***B***. Current density plot (*C*) of neuronal *I*_DR_ from recordings as shown in panel ***A***. Data are presented as mean ± SEM, and ‘n’ numbers for each data group are mentioned within panels. In order to better visualize the changes in *I*_DR_ current density at minimal depolarizing potentials, traces from -60 mV to -10 mV test-potentials are shown with magnification inside the cyan box inset. ***D***, Blockade of currents through Kv2.1 with stromatoxin-1 (ScTx-1; 100 nM, 30 min) occluded the gp120-induced increase in *I*_DR_ current density. Data are presented as mean ± SEM *I*_DR_ current density at -60 mV, -50 mV and -40 mV test-potentials, deduced from the data shown in panel ***C***. *p<0.05 indicates significantly different in comparison to control/untreated conditions at respective test-potentials; ^#^ p<0.05 indicates significantly different in comparison to gp120-treatment conditions at respective test-potentials (one way ANOVA with post-hoc Bonferroni’s correction).

**Figure 5:**
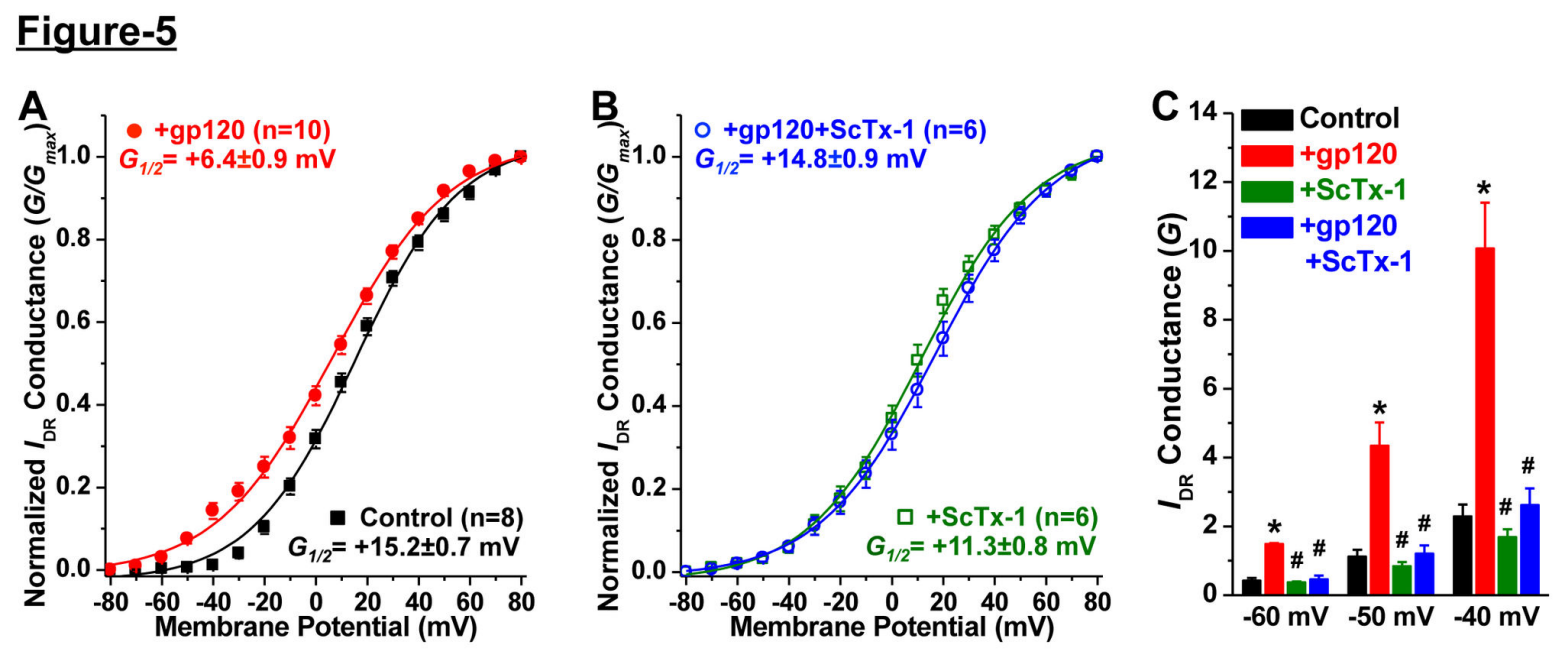
Acute exposure to HIV-1 gp120 enhances neuronal *I*_DR_ conductance via specific modulation of the Kv2.1 channel. *A*, Normalized conductance-voltage relationship of *I*_DR_ in cultured rat hippocampal neurons, showing ~9 mV hyperpolarizing shift in the half-maximal conductance (*G*_*1/2*_; values shown within the panel). *B*, Blockade of currents through Kv2.1 with ScTx-1 (100 nM, 30 min) occluded the gp120-induced increase in *I*_DR_ conductance. Data in panels ***A*** & ***B*** are presented as mean ± SEM and fitted value ± SE of fit for *I*_DR_ conductances, and the ‘n’ numbers for each data group are mentioned within panels. ***C***, Increase in *I*_DR_ conductance upon gp120 exposure of neurons at minimal depolarizing potentials is specific to ScTx-1 sensitive currents. Data are presented as mean ± SEM of actual *I*_DR_ conductances at -60 mV, -50 mV and -40 mV test-potentials for control and gp120 +/- ScTx-1 treatment groups, obtained from the same voltage-clamp recordings used for quantifying normalized conductance-voltage relationships shown in panel ***B***. *p<0.05 indicates significantly different in comparison to control/untreated conditions at respective test-potentials; ^#^ p<0.05 indicates significantly different in comparison to gp120-treatment conditions at respective test-potentials (one way ANOVA with post-hoc Bonferroni’s correction).

Given that acute gp120 treatment led to Kv2.1 channel dephosphorylation and altered channel localization, we next determined the effect of such modifications on the functional properties of the channel. Treatment of cultured rat hippocampal neurons with gp120 (10 nM, 30 min) did not lead to any change in the current density of *I*_DR_ at stronger depolarizing potentials (Figure 4A, C); however, a significant increase in the current density was observed at depolarizing potentials close to the resting membrane potential (Figure 4B-D). Treatment of neurons with ScTx-1 (100 nM, 30 min), a specific inhibitor of Kv2.1 channel at sub-micromolar concentrations [33,34], led to a ~35% reduction in *I*_DR_ density (Figure 4C-D). Importantly, ScTx-1 treatment of gp120-treated neurons attenuated the increased current density that was observed at depolarizing potentials close to the resting membrane potential (Figure 4C-D). Further analysis of the voltage-dependent activation/conductance relationship (*G-V*) revealed that acute gp120 treatment leads to a ~9 mV hyperpolarizing shift in the half-maximal conductance of neuronal *I*_DR_ (*G*_*1/2*_ = +15.17±0.67 mV and +6.39±0.91 mV for control and gp-120-treated neurons, respectively; Figure 5A). ScTx-1 treatment occluded this gp120-induced shift in the half-maximal conductance of neuronal *I*_DR_ (*G*_*1/2*_ = +11.27±0.81 mV and +14.82±0.94 mV for ScTx-1- and ScTx-1+gp-120-treated neurons, respectively; Figure 5B). Furthermore, the gp120-induced increase in *I*_DR_ conductances observed at depolarizing potentials close to the resting membrane potential (-60/-50/-40 mV; Figure 5A-C) was attenuated with ScTx-1 treatment of neurons (Figure 5C). In must be noted here that, although treatment of neurons with ScTx-1 alone shows an increase in the normalized *I*_DR_ conductance at depolarizing potentials close to the resting membrane potential (Figure 5B), there was no change in the total *I*_DR_ conductance at these potentials (Figure 5C). This is consistent with the observation that ScTx-1 treatment leads to a ~35% reduction in peak current density of neuronal *I*_DR_ at +80 mV; however, no significant change in *I*_DR_ density was observed at -60/-50/-40 mV (Figure 4C-D). These observations are in line with prior reports, which clearly and convincingly showed that under physiological conditions and/or constitutively phosphorylated state, Kv2.1 channel conducts no or negligible current at -60/-50/-40 mV [8,21,24,25]. Most importantly, no further changes were observed in the current density and conductance of ScTx-1-resistant *I*_DR_ upon gp120 treatment (Figures 4C-D, 5B-C). This suggests that acute gp120 exposure led to specific enhancement of ScTx-1-sensitive neuronal *I*_DR_, which corresponds to Kv2.1 currents. No significant changes in current density and activation of the fast-activating and inactivating outward K^+^ currents (*I*_A_) were observed upon acute gp120 treatment of neurons (not shown). These results indicate that acute treatment of neurons with gp120 leads to a significant enhancement of *I*_DR_ conductance at near resting membrane potentials, by specifically up-regulating Kv2.1 channel function, a modification which could profoundly suppress cellular excitability, thereby providing a protective mechanism for neurons.

### Acute HIV-1 gp120 exposure protects neurons from non-apoptotic cell death via complex signaling involving CCR5/CXCR4, calcineurin and Kv2.1

**Figure 6:**
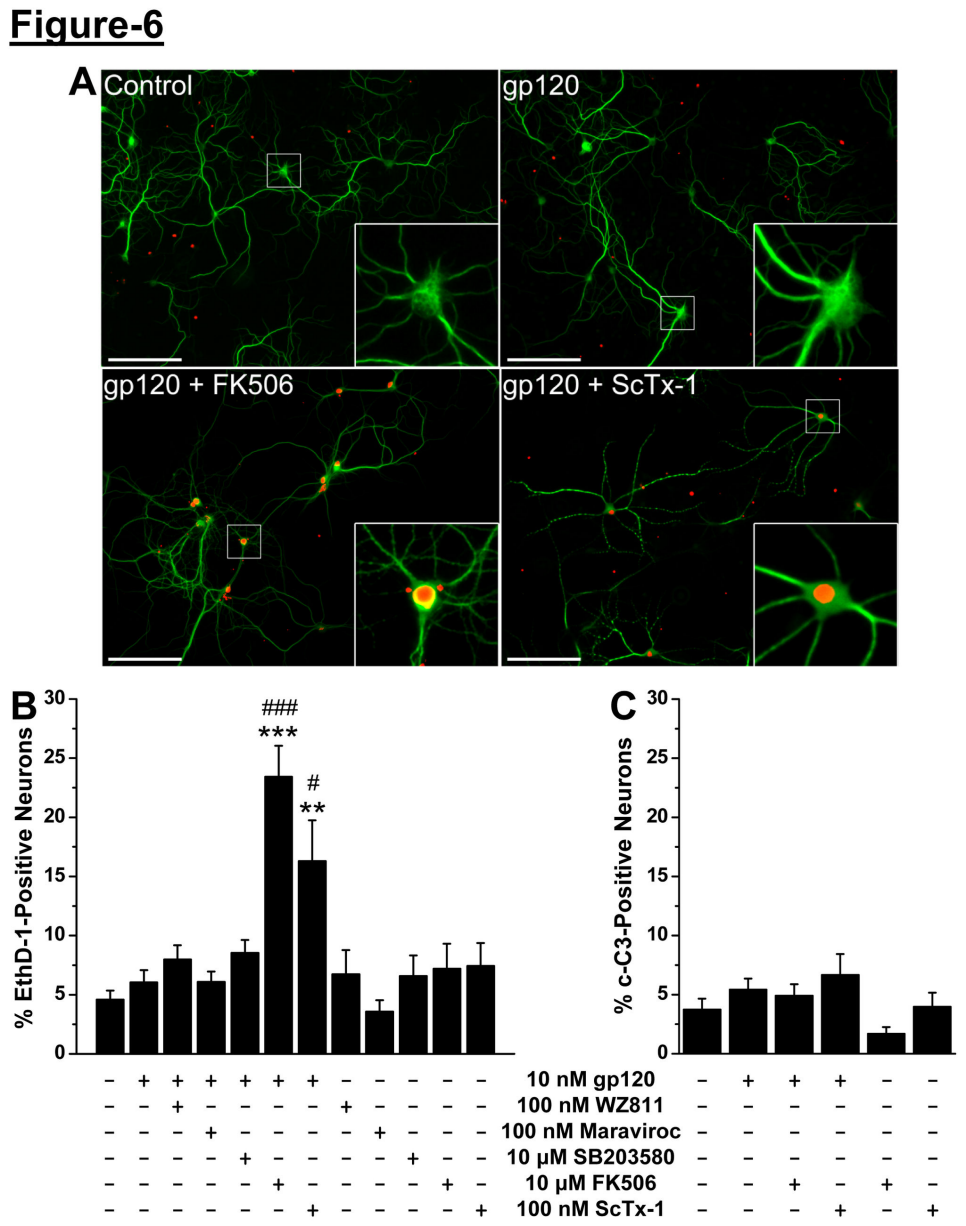
Acute gp120 treatment provides neuroprotection via calcineurin-dependent dephosphorylation of Kv2.1. ***A***, Representative images of MAP2-positive cultured rat hippocampal neurons (green) labeled with ethidium homodimer (EthD-1), which is an indicator of dead or dying cells. Under control and gp120 treatment (10 nM, 30 min) conditions (top row), the majority of MAP2-positive cells were EthD-1 negative. However, when gp120 was applied in the presence of the calcineurin inhibitor FK506 (10 µM) or Kv2.1-blocking toxin ScTx-1 (100 nM; bottom row), MAP2-positive cells with EthD-1-positive nuclei became more prevalent. ***B***, Quantification of (EthD-1)-positive neurons (MAP2-positive) following different drug treatment conditions as detailed in the bottom of the graph. Acute gp120 (10 nM, 30 min), only upon co-application with FK506 (10 µM) or ScTx-1 (100 nM) led to a significant increase in the extent of neuronal death. No change in the extent of neuronal death was observed upon co-application of gp120 either with CXCR4 inhibitor WZ811 (WZ811) or CCR5 inhibitor Maraviroc (100 nM) or p38 MAPK inhibitor SB203580 (10 µM). ***C***, Quantification of cleaved-caspase-3 (c-C3)-positive neurons (MAP2-positive) as a measure of cells undergoing apoptotic death, following different drug treatment conditions as detailed in the bottom of the graph. None of the treatment conditions led to a significant increase in the number of c-C3-positive neurons. Treatment of neurons with the inhibitors alone did not alter the percentage of EthD-1-positive (***B***) and c-C3-positive neurons (***C***). For both the panels data are presented as mean ± SEM. n = 1006 (control/untreated), 909 (gp120 alone), 684 (WZ811+gp120), 665 (Maraviroc+gp120), 655 (SB230580+gp120), 886 (FK506+gp120), 839 (ScTx-1+gp120), 960 (WZ811 alone), 669 (Maraviroc alone), 1023 (SB203580 alone), 1134 (FK506 alone), and 681 (ScTx-1 alone) treatment groups, obtained from ≥4 batches of cultured rat hippocampal neurons. **p<0.01 and ***p<0.001 indicate significantly different in comparison to control/untreated conditions; ^#^ p<0.05 and ^# # #^ p<0.001 indicate significantly different in comparison to gp120-treatment conditions, one way ANOVA with post-hoc Bonferroni’s correction).

Since acute gp120 exposure leads to dephosphorylation of Kv2.1 and enhanced voltage-dependence of channel activation, we next tested whether these changes could provide neuroprotection. Application of gp120 (10 nM, 30 min) led to no significant increase in the number of neurons with ethidium homodimer-1 (EthD-1)-positive nuclei, an indicator of dead or dying cells (Figure 6A-B), which was not altered by pre-application of CCR5 or CXCR4 inhibitors maraviroc and WZ811 (100 nM each, 10 min; Figure 6B). Since, treatment with gp120 alone did not induce any cell death, it was expected that application of gp120 with the inhibitors of CCR5 or CXCR4 would also not induce any cell death, because effective blockade of CCR5/CXCR4 activation would presumably mimic a control/un-treated like condition for neurons. Furthermore, pre-application of the inhibitor of pro-apoptotic p38 MAPK, SB203580 (1 µM, 10 min), with gp120 also did not influence neuronal cell death (Figure 6B). However, pre-application of either the calcineurin inhibitor FK506 (10 µM, 10 min) or the Kv2.1 channel-blocking toxin ScTx-1 (100 nM, 10 min) with gp120 led to a significant increase in the extent of neuronal death (Figure 6A-B). None of the inhibitors listed above had any influence on neuronal death, when applied in the absence of gp120 (Figure 6B). These observations suggest that acute gp120-induced Ca^2+^/calcineurin-dependent modulation of Kv2.1 specifically provides homeostatic protection to neurons. However, under conditions of blockade of calcineurin activity or Kv2.1 channel function, neurons lack such homeostatic protective mechanisms against gp120-induced elevation in [Ca^2+^]_i_ and excitotoxic cell death, thereby suggesting the critical role of modulated Kv2.1 channels in this context.

We next determined whether the blockade of calcineurin activity and Kv2.1 function that leads to gp120-induced neuronal death was apoptotic in nature or not. We quantified the number of neurons with cleaved caspase-3 (c-C3) stained nuclei, which provides an indicative measure of neuronal apoptosis [35,36]. Acute gp120 application (10 nM, 30 min) did not lead to any significant increase in the number of nuclear c-C3 stained neurons without or with the co-application of FK506 or ScTx-1 (Figure 6C). None of the inhibitors listed above had any influence on neuronal death/apoptosis, when applied in the absence of gp120 (Figure 6B-C). These results suggest that acute gp120 could induce non-apoptotic, but presumably excitotoxic neuronal death upon inhibition of calcineurin or Kv2.1 channel activity.

## Discussion

Despite the many clinical advances made in antiretroviral therapy, HIV-associated neurodegeneration continues to pose a serious and growing medical problem [2,3]. The viral coat glycoprotein gp120 is known to be a major component of the neuronal death associated with HAND [2,4,8,37,38], but a comprehensive understanding of the ‘acute phase’ response of neurons to HIV and, more specifically, gp120 is currently lacking. Our results elucidate a novel mechanism whereby neurons are able to activate their intrinsic homeostatic/protective pathway in response to acute gp120 exposure, which could otherwise lead to excitotoxic death due to [Ca^2+^]_i_ elevation. The Ca^2+^/calmodulin-dependent protein phosphatase calcineurin mediates this process by dephosphorylating the major somatodendritic Kv channel Kv2.1. Aside from being associated with a change in the subcellular distribution of the channel, this dephosphorylation also coincides with a change in the channel’s voltage-dependent activation properties, such that current densities at voltages close to the resting membrane potential are significantly increased. This results in a protective reduction in neuronal excitability, a point emphasized by the fact that, when this homeostatic response is inhibited (i.e. by inhibiting calcineurin activity or blocking the Kv2.1 channel activation itself), neurons are no longer able to maintain normal levels of excitability and undergo excitotoxic/necrotic cell death.

The ability of gp120 to elicit [Ca^2+^]_i_ elevation in neurons is potentially secondary to the release of neurotransmitters and/or inflammatory mediators from nearby glial cells and other neurons [5,7,11,13,14]. This possibility is supported by our observation that gp120-induced dephosphorylation of Kv2.1 could be prevented by MK801 (Figure 2), a GluN receptor antagonist. However, this Ca^2+^ flux could also be due directly to engagement of chemokine co-receptors CXCR4 and CCR5, receptors that are both known to predominantly couple to Gα_i_ subunits [14], the associated Gβγ subunit of which can induce Ca^2+^ release via activation of phospholipase (PLC)-β-mediated hydrolysis of phosphatidyl inositol bis-phosphate (PIP_2_) [39]. Additionally, both CCR5 and CXCR4 have also been shown to couple to Gα_q_ subunits, which lead to PLCβ activation, PIP_2_ hydrolysis and Ca^2+^ release from intracellular stores [40].

It is an interesting observation that, despite the ability of both chemokine receptor blockers to prevent gp120-induced Kv2.1 dephosphorylation and altered localization of the channel protein, the endogenous chemokine ligand for CCR5, RANTES, did not replicate the gp120-induced changes in Kv2.1 (Figure 2B). However, the endogenous chemokine ligand for CXCR4, SDF-1α, is capable of elevating intracellular Ca^2+^ levels and Kv2.1 dephosphorylation and altered channel localization in neurons (Figure 2B and [8]). This suggests that, although interaction with both chemokine receptors is required for full gp120 binding and subsequent activation of downstream signaling, the elevation in [Ca^2+^]_i_ levels and activation of calcineurin in neurons is contributed predominantly by CXCR4-mediated signaling events. Whilst there is no direct evidence from our data to confirm or refute this hypothesis, it is intriguing to note that CCR5 signaling downstream of gp120 has been reported to serve a protective role by reducing CXCR4-mediated [Ca^2+^]_i_ elevation [7]. Also, the magnitude of RANTES-induced elevation in [Ca^2+^]_i_ in rodent hippocampal neurons has been reported to be several-fold less and mostly transient and oscillatory in nature, as compared to gp120- and SDF-1α-induced [Ca^2+^]_i_ elevation [10]. Thus, further thorough investigation is required to determine whether CCR5 is only required to constitute a co-receptor complex with CXCR4, in order to facilitate gp120 binding, with the downstream signaling being predominantly mediated via CXCR4 activation. Also, it is important to determine the precise coupling of CCR5 and CXCR4 under homomeric and co-receptor configurations with Gα_i_ and/or Gα_q_ subunits and specific downstream signaling in neurons and microglia/astrocytes. This would provide better explanations for the distinct roles of these chemokine receptors, upon physiological activation by chemokine activators versus pathological activation by infectious agents/factors such as HIV-1 gp120.

Whilst it is clear that gp120 is clearly a major pathophysiological mediator of HAND, we cannot rule out potential effects of other HIV components, such as Tat (‘trans-activator of transcription’) and Nef (‘negative factor’), with which excitotoxic effects have also been reported [5,13,18,38,41-43]. It is an intriguing possibility, but it remains to be confirmed whether HIV-1 Tat and Nef exposure causes changes in Kv2.1 channel properties similar to those seen with gp120. Furthermore, Kv2.1 is not the sole arbiter of overall neuronal excitability, nor is it likely to be the only Kv channel whose properties are altered by neuro-HIV infection. A recent study indicates that gp120 causes an enhancement of *I*_A_ currents in cultured rat cortical neurons via CXCR4 signaling, a change that was also suggested to underlie neuronal apoptosis following prolonged exposure to gp120 [44]. This transient *I*_A_ current in neurons is contributed by several Kv channel subfamily members, including Kv1.4 and α-subunits belonging to the Kv4 family [22]. However, we did not observe any significant change in *I*_A_ currents in response to acute gp120 treatment of cultured rat hippocampal neurons, rather a pronounced enhancement of *I*_DR_ component that is specifically mediated by the Kv2.1 channel. Similar enhancement of Kv1.3-based *I*_DR_ current density and voltage-dependent activation gating via CXCR4 and downstream protein kinase A signaling were observed in cultured rat microglia upon gp120 treatment [45]. Given the widespread expression, specialized subcellular localization and dynamic modulation of Kv2.1 channel’s voltage-dependent activation properties in mammalian central neurons, it is our contention that gp120-induced enhancement of Kv2.1 activation constitutes the predominant source of neuroprotective outward K^+^ current in the acute stages of neuro-HIV infection. Targeting such intrinsic cellular mechanisms might provide effective neuroprotection against HAND, which has always been positively debated [46,47].

Therefore, gp120-induced rapid dephosphorylation of Kv2.1, as it can occur within minutes, can be thought of as an ‘acute-phase response’ that acts to limit excitability of the neuron and postsynaptic neurons in the surrounding area when conditions are such that high-frequency firing is likely to occur. Our previous observations clearly showed that glutamate treatment of hippocampal neurons led to Ca^2+^/calcineurin-mediated dephosphorylation of Kv2.1, which resulted in hyperpolarizing shifts in the voltage-dependent activation/conductance of Kv2.1 currents [21]. We have also shown that these changes in Kv2.1 lead to suppression of neuronal firing frequencies in current-clamp electrophysiological assays on cultured hippocampal neurons [21]. Both spontaneous and current-injected action potential firings were significantly reduced upon acute Ca^2+^/calcineurin-mediated dephosphorylation of Kv2.1. Since acute exposure of gp120 induces similar changes in Kv2.1 phosphorylation status and voltage-dependent activation/conductance properties in hippocampal neurons, it is reasonable to predict a significant reduction in neuronal firing frequencies under such conditions. We have demonstrated the short-term effectiveness of this homeostatic response in terms of neuroprotection, yet we must reconcile these observations with the overt apoptotic neuronal death seen in HAND. Although acute exposure to HIV-1 gp120 leads to the activation of multiple CXCR4/CCR5-mediated downstream signaling events, the Ca^2+^/calcineurin/Kv2.1 signaling axis is the critical determinant of acute homeostatic survival responses in neurons. What remains largely unexplored is exactly how, when and for what reasons this protective mechanism fails, and neurons undergo apoptotic cell death, questions our future research aims to address. Developing our understanding of these mechanisms and their interplay will provide us with novel therapeutic opportunities for HAND, and perhaps other neurodegenerative disorders.
